# Divergent and non-parallel evolution of MHC IIB in the Neotropical Midas cichlid species complex

**DOI:** 10.1186/s12862-022-01997-9

**Published:** 2022-04-01

**Authors:** Seraina E. Bracamonte, Melinda J. Hofmann, Carlos Lozano-Martín, Christophe Eizaguirre, Marta Barluenga

**Affiliations:** 1 Museo Nacional de Ciencias Naturales, CSIC, José Gutiérrez Abascal 2, 28006 Madrid, Spain; 2 School of Biological and Chemical Sciences, Queen Mary University of London, Mile End Road, London, E1 4NS UK

**Keywords:** Major histocompatibility complex, Ecological divergence, Adaptive radiation, *Amphilophus* species complex

## Abstract

**Background:**

Ecological diversification is the result of divergent natural selection by contrasting habitat characteristics that favours the evolution of distinct phenotypes. This process can happen in sympatry and in allopatry. Habitat-specific parasite communities have the potential to drive diversification among host populations by imposing selective pressures on their host's immune system. In particular, the hyperdiverse genes of the major histocompatibility complex (MHC) are implicated in parasite-mediated host divergence. Here, we studied the extent of divergence at MHC, and discuss how it may have contributed to the Nicaraguan Midas cichlid species complex diversification, one of the most convincing examples of rapid sympatric parallel speciation.

**Results:**

We genotyped the MHC IIB for individuals from six sympatric Midas cichlid assemblages, each containing species that have adapted to exploit similar habitats. We recovered large allelic and functional diversity within the species complex. While most alleles were rare, functional groups of alleles (supertypes) were common, suggesting that they are key to survival and that they were maintained during colonization and subsequent radiations. We identified lake-specific and habitat-specific signatures for both allelic and functional diversity, but no clear pattern of parallel divergence among ecomorphologically similar phenotypes.

**Conclusions:**

Colonization and demographic effects of the fish could have contributed to MHC evolution in the Midas cichlid in conjunction with habitat-specific selective pressures, such as parasites associated to alternative preys or environmental features. Additional ecological data will help evaluating the role of host–parasite interactions in the Midas cichlid radiations and aid in elucidating the potential role of non-parallel features differentiating crater lake species assemblages.

**Supplementary Information:**

The online version contains supplementary material available at 10.1186/s12862-022-01997-9.

## Background

Diversification into ecologically and morphologically distinct forms upon colonization of novel habitats is the result of natural selection, and this can lead to convergent evolution of similar forms in similar but geographically separated habitats [1–4]. Both species interactions and abiotic environmental properties of the habitat contribute to this process [5]. Parasites are ubiquitous biotic agents [6], that are recognized as powerful drivers of diversification [7–10]. Experiments on antagonistic coevolution of hosts and parasites evidenced rapid evolutionary changes and divergence of host populations [11–13].

Parasites interact directly with the host immune system, and therefore, variability in parasite communities leaves detectable signatures in the immune response and its underlying genetic basis [14]. The immunogenes of the major histocompatibility complex (MHC) play a crucial role in the adaptive immune response of jawed vertebrates [15, 16]. They are hyperdiverse and tend to segregate among populations [17–19]. Thus, MHC genes are excellent candidates for studying the link between parasites, immunogenetic adaptation, and host diversification [7, 20, 21]. MHC genes encode cell-surface glycoproteins that bind peptide fragments derived from parasites and present them to T-cells, thereby activating the adaptive immune response [16]. Broadly, intracellular parasites (e.g. viruses) are recognized by MHC class I, while MHC class II recognizes extracellular parasites (e.g. bacteria or helminths). MHC class I molecules are heterodimers consisting of two polypeptide chains, Iα and a β2-microglobulin. MHC class II molecules are also heterodimers, with two homologous chains IIα and IIβ, encoded by MHC IIA and MHC IIB genes, respectively. MHC genes are the most polymorphic vertebrate genes known to date [16, 22], with variation concentrated in the antigen-binding site, the region determining specificity of the molecule [23, 24]. For MHC class II, polymorphism is mostly contained by exon 2 of MHC IIB. Experimental evidence and theoretical models suggest that the high levels of polymorphism may be the result of balancing selection mediated by parasites [25, 26]. Furthermore, there is increasing evidence that MHC also interacts with an individual’s microbiota [27, 28].

Spatial variation in selection on the MHC can lead to a patchwork of immunogenetic divergence and local adaptation among populations occupying different habitats [29]. Divergence may then be reinforced by MHC-assortative mate choice to increase resistance and attain immunogenetic optimality of offspring [17, 20, 30, 31]. Similar parasite and microbial communities within habitat types associated to alternative preys or substrates are expected to lead to similarities at MHC and parallel divergence among habitat types, although empirical evidence is inconclusive. Some studies in the freshwater fish model three-spined stickleback indeed found repeated parallel divergence at MHC among ecotypes [17, 32, 33]; however, other studies in sticklebacks and African cichlids identified population-specific MHC pools and associated divergence [18, 34]. On the other hand, divergent ecotypes can retain similar MHC pools among very contrasting habitats, as shown in the livebearing freshwater fish *Poecilia mexicana* [35]. Hence, predicting the evolutionary outcome of processes on MHC allele segregation is difficult.

Cichlid fish are textbook examples of adaptive radiations, forming the most species-rich and phenotypically diverse clade of teleosts [36]. Ecologically and morphologically similar species are frequently found within and among lakes, and even between continents [3, 37, 38]. An increasing number of studies investigates the importance of parasites in host diversification [39–43] and investigates their ecological interaction ([43], Santacruz et al., [44]). Cichlids possess an extremely large number of MHC class II genes, up to 13 loci per haplotype [45], and show high allelic diversity [46–48], a feature that could facilitate immune specialization [13], enable habitat and trophic adaptation [32], and influence assortative mating [49], which may ultimately contribute to speciation.

**Fig. 1:**
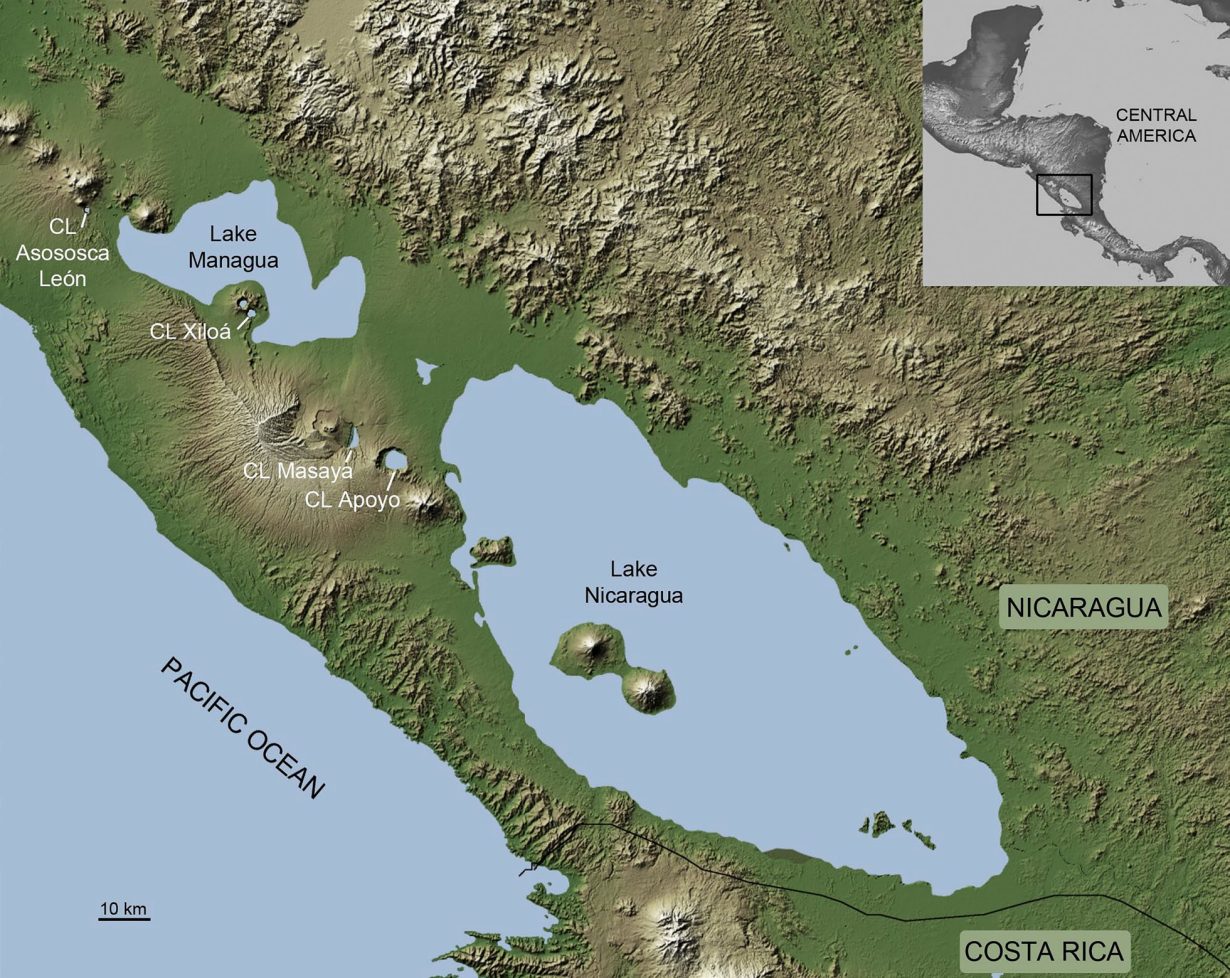
Map of the Nicaraguan great lakes region showing sampled water bodies (modified from NASA Photojournal PIA03364)

The Neotropical Midas cichlid, *Amphilophus* spp., has recently colonized several isolated volcanic crater lakes, independently and asynchronously, from source populations in the tectonic great Nicaraguan lakes, Managua and Nicaragua [50–52] (Fig. 1). Colonization was followed by ecological divergence and sympatric speciation producing closely related species assemblages within each lake [51–54]. Convergent phenotypes can be found along different ecological axes among lakes (e.g. benthic *vs* limnetic in crater lakes (CL) Apoyo and Xiloá; [55, 56], or the rocky (exploited by thick-lipped fish) vs sandy (exploited by normal-lipped fish) substrates in the great lakes; [57, 58]) that are more closely related to species within their respective lakes that to similar phenotypes in other lakes [51, 54]. These ecomorphotypes have been described as distinct species in some lakes [59, 60] while in others, until more work is done, they are still considered to form single polymorphic populations [54].

Here we investigate the contribution of MHC to repeated and parallel divergence of the Midas cichlid in six sympatric assemblages inhabiting isolated crater lakes. First, we characterized the sequence diversity of MHC class IIB alleles in the Midas cichlid species complex across Nicaraguan lakes using high-throughput sequencing. We identified sites under positive selection in the MHC alleles, inferred their phylogenetic relationship, and assigned supertypes of functionally similar alleles. Second, we identified divergence at MHC IIB within the species complex. We tested for differences in sequence diversity among populations, lakes, and habitats. We identified the distribution of alleles among fish in different lakes, populations or species within lakes, and fish in shared habitats across lakes, and tested for divergence in allele and supertype pools. We then identified alleles and supertypes that contributed most to this divergence.

## Results

### Molecular sequence analyses

We analysed a total of 287 individuals from six sympatric assemblages, each containing species that have adapted to exploiting benthic-limnetic or sandy-rocky habitats (Table 1). A total of 4,158,917 paired-end reads passed the initial quality control, with a mean of 14,441 reads per sample (range: 1713–61,269). These were clustered into 152 unique MHC class II exon 2 alleles, 130 new and 22 corresponding to previously identified alleles [47]. Alleles identified in this study were named ac001 to ac130. Two alleles were excluded from further analyses: the putative non-classical allele Amci-DXB*000101 (see [47]), and one allele with premature stop codons due to a 1 bp deletion. One allele had a 3 bp deletion, and since this did not result in premature stop codons, it was kept in the analyses (Additional file 1: Fig. S1). The resulting 150 alleles translated into 146 unique amino acid sequences. The fragment length was 142 bp of which 97 sites were variable. In the translated amino acid sequences, 35 of 47 sites were variable (Additional file 1: Fig. S2). The nucleotide p-distance among all sequences (π) ± SE was 0.20 ± 0.018, the amino acid p-distance (aa p-dist) was 0.33 ± 0.042, the dN was 0.24 ± 0.042, and the dS was 0.21 ± 0.053.

**Table 1:** Midas cichlid populations used in this study with their characteristic morphology and preferred habitat

Lake	Lake type	Population	Morphotype	Habitat	n
Nicaragua	Tectonic	*A. citrinellus*	High-bodied	Shallow benthic	24
		*A. labiatus*	Thick-lipped	Rocky	25
Managua	Tectonic	*A. citrinellus*	High-bodied	Shallow benthic	20
		*A. labiatus*	Thick-lipped	Rocky	4
Masaya	Crater	*A. cf. citrinellus*	High-bodied	Shallow benthic	27
		*A. cf. labiatus*	Thick-lipped	Rocky	18
Asososca León	Crater	*A. citrinellus* f. benthic	High-bodied	Shallow benthic	23
		*A. citrinellus* f. limnetic	Long-bodied	Limnetic	20
Apoyo	Crater	*A. astorquii*	High-bodied	Shallow benthic	20
		*A. chancho*	High-bodied	Deep benthic	21
		*A. zaliosus*	Long-bodied	Limnetic	21
Xiloá	Crater	*A. amarillo*	High-bodied	Shallow benthic	22
		*A. xiloaensis*	High-bodied	Deep benthic	11
		*A. sagittae*	Long-bodied	Limnetic	31
Total					287

n, number of sampled individuals per population

**Fig. 2:**
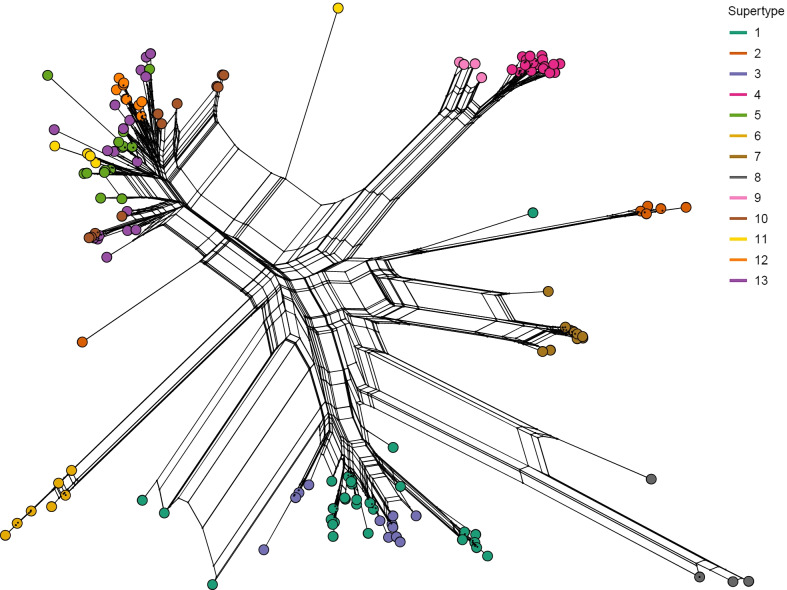
Splits network of the phylogenetic relationship of Midas cichlid MHC IIB alleles. Colours indicate the supertype to which each allele was assigned

A codon-based Z-test of selection did not provide evidence for gene-wide positive selection on alleles over evolutionary times (Z = 0.8, p = 0.2). However, codon models allowing for site-specific or branch-site-specific positive selection provided a better fit for the data than models without positive selection (Table 2). Selection models implemented in CodeML identified 13 positively selected sites (PSS), MEME identified 16 PSS, and analyses with MrBayes identified 10 PSS. Eight sites under positive selection were identified by all methods and an additional four sites were identified by two methods (Additional file 1: Fig. S2). Reducing alleles to PSS resulted in 115 unique amino acid sequences which clustered in 13 supertypes of putatively functionally similar alleles. The number of alleles per supertype varied from 4 to 29 (Fig. 2).

**Table 2:** Positive site-specific selection identified with CodeML models, BUSTED and MEME, and MrBayes

Model	logL	Parameter estimates	PSS
M1a^a^	− 3068.35	p_0_ = 0.606, p_1_ = 0.394 ω_0_ = 0.128, ω_1_ = 1	NA
M2a^a^	− 2985.41	p_0_ = 0.379, p_1_ = 0.366, p_2_ = 0.255 ω_0_ = 0.073, ω_1_ = 1, ω_2_ = 4.138	1, 3, 10, 12, 13, 22, 28, 35, 40, 42, 44, 45, 46
M7^a^	− 3052.97	p = 0.171, q = 0.184	NA
M8^a^	− 2984.37	p_0_ = 0.749, p_1_ = 0.251, p = 0.194 q = 0.219, ω = 3.767	1, 3, 10, 12, 13, 22, 28, 35, 40, 42, 44, 45, 46
Constrained^b^	− 2885.7	p_1_ = 0.369, p_2_ = 0, p_3_ = 0.631 ω_1_ = 0, ω_2_ = 0.54, ω_3_ = 1	NA
Unconstrained^b^/MEME	− 2805.1	p_1_ = 0.351, p_2_ = 0.543, p_3_ = 0.106 ω_1_ = 0.37, ω_2_ = 0.37, ω_3_ = 43.67	1, 3, 5, 12, 13, 16, 21, 22, 35, 36, 39, 42, 44, 45, 46, 47
MrBayes		p_−_ = 0.356, p_N_ = 0.359, p_+_ = 0.284 ω_−_ = 0.082, ω_N_ = 1, ω_+_ = 4.577	1, 3, 13, 22, 28, 35, 36, 42, 45, 46

logL, log-likelihood value; PSS, positively selected sites; NA, not allowed; PSS indicated for the unconstrained model are identified with MEME. Model parameters are: M1a and M2a: p_0_ = proportion of sites with 0 < ω_0_ < 1, p_1_ = proportion of sites with ω_1_ = 1, p_2_ = proportion of sites with ω_2_ > 1; M7 and M8: p, q = β distribution parameters, p_0_ = proportion of sites with ω within the β distribution, p_1_ = proportion of sites with ω > 1; constrained and unconstrained: p_1_, p_2_ = proportion of sites with 0 ≤ ω_1_ ≤ ω_2_ ≤ 1, p_3_ = proportion of sites with ω_3_ = 1 (constrained) or ω_3_ > 1 (unconstrained); MrBayes: p_−_ = proportion of sites with 0 ≤ ω_−_ < 1, p_N_ = proportion of sites with ω_N_ = 1, p_+_ = proportion of sites with ω_+_  > 1

^a^CodeML models

^b^BUSTED models

A split network grouped alleles into seven major clusters, though they were only weakly delimited (Fig. 2). Bayesian phylogenetic inference also indicated that alleles fall into seven clusters with posterior probabilities > 80 (Additional file 1: Fig. S3). All major clusters but one were composed of one or two supertypes, which were largely monophyletic. The last cluster consisted of three paraphyletic supertypes.

### Population divergence analyses

**Fig. 3:**
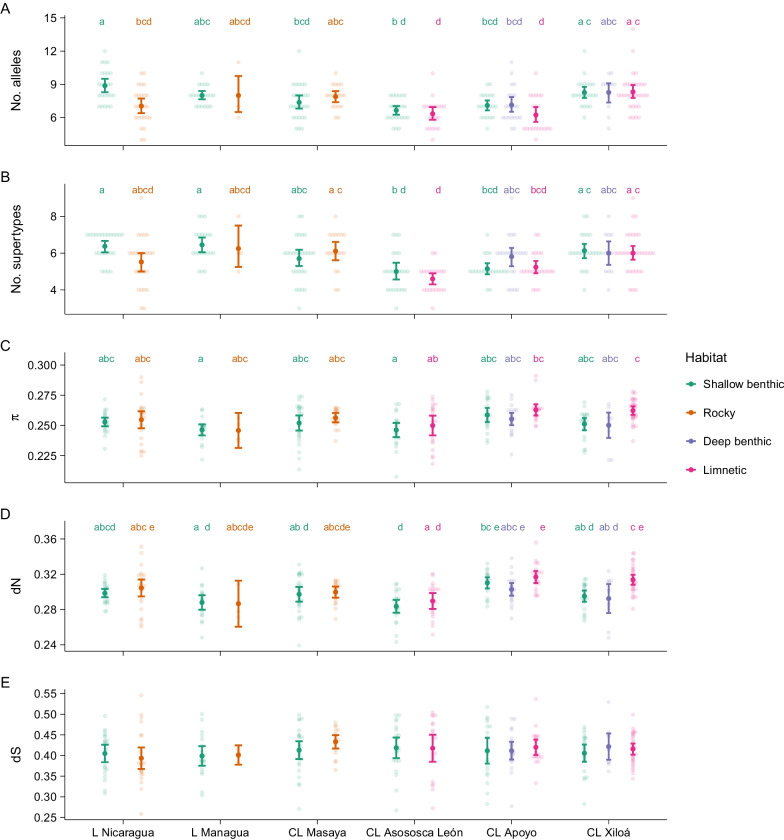
Within-individual sequence diversity indices of MHC IIB by lake and habitat (mean ± 95% CI). **A** Number of alleles, **B** number of supertypes, **C** nucleotide p-distance π, **D** dN, **E** dS. Populations are from left to right: *A. citrinellus*, *A. labiatus*, *A. citrinellus*, *A. labiatus*, *A. c.f. citrinellus*, *A. c.f. labiatus*, *A. citrinellus* f. benthic, *A. citrinellus* f. limnetic, *A. astorquii*, *A. chancho*, *A. zaliosus*, *A. amarillo*, *A. xiloaensis*, *A. sagittae*. Light coloured points give the values for each individual

The mean ± SE number of alleles per individual was 7.5 ± 0.1 (range: 4–14), with a minimum of 6.2 ± 0.35 for *A. zaliosus* in CL Apoyo and a maximum of 8.9 ± 0.30 for *A. citrinellus* in L Nicaragua (Table 3, Fig. 3A). The mean ± SE number of supertypes per individual was 5.7 ± 0.07. It was lowest in *A. citrinellus* f. limnetic in CL Asososca León and highest in *A. citrinellus* in L Nicaragua and L Managua (Table 3, Fig. 3B). We additionally calculated four diversity indices for each individual. Mean ± SE π, aa p-dist, dN, and dS per individual were 0.254 ± 0.001, 0.392 ± 0.001, 0.299 ± 0.001, and 0.411 ± 0.003, respectively. Π, aa p-dist, and dN were lowest in *A. citrinellus* f. benthic in CL Asososca León and highest in *A. zaliosus* in CL Apoyo and *A. sagittae* in CL Xiloá (Table 3, Fig. 3C, D). DS was lowest in *A. labiatus* in L Nicaragua and highest in *A. cf. labiatus* in CL Masaya (Table 3, Fig. 3E). Aa p-dist and dN were highly correlated (Pearson’s R = 0.94, p-value < 0.001), hence only dN was analysed further. Within-individual sequence diversity indices except dS differed significantly among populations (Table 4). The number of alleles and the number of supertypes per individual differed significantly among lakes but not among habitats. However, there was a significant interaction between lake and habitat. Within-individual π and dN differed significantly among lakes and habitats. There was no significant difference in dS among lakes or habitats (Table 4).

**Table 3:** Diversity indices for populations, lakes, and averaged across all samples of the Midas cichlid populations

	n	No. alleles	No. priv. alleles	No. supertypes	Mean no. alleles	Mean no. supertypes	π	aa p-dist	dN	dS
L Nicaragua	49	66	14	13	7.9 ± 0.26	5.9 ± 0.17	0.254 ± 0.002	0.397 ± 0.003	0.299 ± 0.003	0.396 ± 0.008
* A. citrinellus*	24	50	8	13	8.9 ± 0.30	6.4 ± 0.16	0.253 ± 0.002	0.395 ± 0.003	0.295 ± 0.003	0.400 ± 0.011
* A. labiatus*	25	38	4	11	7.0 ± 0.33	5.5 ± 0.28	0.255 ± 0.003	0.400 ± 0.005	0.303 ± 0.005	0.392 ± 0.013
L Managua	24	66	22	13	8.0 ± 0.22	6.4 ± 0.20	0.246 ± 0.002	0.382 ± 0.004	0.288 ± 0.003	0.399 ± 0.009
* A. citrinellus*	20	55	19	13	8.0 ± 0.19	6.4 ± 0.21	0.246 ± 0.002	0.382 ± 0.004	0.288 ± 0.004	0.399 ± 0.011
* A. labiatus*	4	23	3	10	8.0 ± 1.08	6.2 ± 0.63	0.246 ± 0.005	0.384 ± 0.009	0.287 ± 0.008	0.401 ± 0.007
CL Masaya	45	46	3	12	7.6 ± 0.21	5.9 ± 0.18	0.254 ± 0.002	0.392 ± 0.003	0.298 ± 0.003	0.421 ± 0.007
* A. cf. citrinellus*	27	40	0	12	7.4 ± 0.30	5.7 ± 0.24	0.252 ± 0.003	0.391 ± 0.004	0.297 ± 0.004	0.413 ± 0.010
* A. cf. labiatus*	18	43	1	12	7.9 ± 0.25	6.1 ± 0.25	0.256 ± 0.002	0.394 ± 0.003	0.300 ± 0.003	0.433 ± 0.008
CL Asososca León	43	27	7	10	6.5 ± 0.18	4.8 ± 0.15	0.248 ± 0.002	0.377 ± 0.003	0.286 ± 0.003	0.418 ± 0.010
* A. citrinellus* f. benthic	23	23	2	9	6.7 ± 0.21	5.0 ± 0.23	0.246 ± 0.003	0.374 ± 0.004	0.284 ± 0.004	0.419 ± 0.012
* A. citrinellus* f. limnetic	20	24	2	10	6.3 ± 0.30	4.6 ± 0.17	0.250 ± 0.004	0.381 ± 0.004	0.290 ± 0.004	0.418 ± 0.016
CL Apoyo	62	47	16	11	6.8 ± 0.19	5.4 ± 0.12	0.259 ± 0.001	0.400 ± 0.002	0.310 ± 0.002	0.414 ± 0.007
* A. astorquii*	20	36	9	10	7.1 ± 0.24	5.2 ± 0.15	0.259 ± 0.003	0.399 ± 0.004	0.310 ± 0.003	0.412 ± 0.015
* A. chancho*	21	28	1	10	7.1 ± 0.35	5.8 ± 0.26	0.255 ± 0.002	0.396 ± 0.003	0.303 ± 0.003	0.412 ± 0.010
* A. zaliosus*	21	22	2	10	6.2 ± 0.35	5.2 ± 0.18	0.263 ± 0.002	0.404 ± 0.003	0.317 ± 0.003	0.420 ± 0.009
CL Xiloá	64	68	17	13	8.3 ± 0.19	6.0 ± 0.13	0.256 ± 0.002	0.396 ± 0.002	0.304 ± 0.002	0.413 ± 0.005
* A. amarillo*	22	46	7	13	8.3 ± 0.26	6.1 ± 0.20	0.251 ± 0.002	0.389 ± 0.003	0.295 ± 0.003	0.406 ± 0.010
* A. xiloaensis*	11	32	4	11	8.3 ± 0.49	6.0 ± 0.36	0.250 ± 0.005	0.386 ± 0.007	0.292 ± 0.007	0.422 ± 0.014
* A. sagittae*	31	31	3	12	8.3 ± 0.30	6.0 ± 0.21	0.262 ± 0.002	0.404 ± 0.003	0.314 ± 0.003	0.416 ± 0.007
Total	287	150		13	7.5 ± 0.10	5.7 ± 0.07	0.254 ± 0.001	0.392 ± 0.001	0.299 ± 0.001	0.411 ± 0.003

n, number of individuals; no. alleles, total number of different alleles; no. priv. alleles, number of private alleles; no. supertypes, total number of different supertypes; mean no. alleles, mean ± SE number of alleles per individual; mean no. supertypes, mean ± SE number of supertypes per individual; π, mean ± SE nucleotide p-distance; aa p-dist, mean ± SE amino acid p-distance; dN, mean ± SE dN; dS, mean ± SE dS

**Table 4:** Model statistics for sequence diversity indices, allele pools, and supertype pools across Midas cichlid populations

Predictor	df	No. alleles		No. supertypes		π			dN			dS			Allele pools		Supertype pools	
		Chisq	p-value	Chisq	p-value	Sum Sq	F	p-value	Sum Sq	F	p-value	Sum Sq	F	p-value	Dev	p-value	Dev	p-value
Population	13	89.338	**< 0.001**	74.892	**< 0.001**	0.008	3.877	**< 0.001**	0.03	7.306	**< 0.001**	0.03	0.769	0.693	1858.01	**0.001**	472.26	**0.001**
Residuals	273					0.043			0.08			0.736						
Lake	5	63.275	**< 0.001**	54.005	**< 0.001**	0.005	5.768	**< 0.001**	0.019	12.909	**< 0.001**	0.015	1.139	0.340	2074.59	**0.001**	334.51	**0.001**
Habitat	3	7.847	**0.049**	4.67	0.198	0.003	5.506	**0.001**	0.008	8.761	**< 0.001**	0.001	0.181	0.909	694.91	**0.001**	45.20	**0.024**
Lake × Habitat	5	19.052	**0.002**	11.852	**0.037**	0.001	0.633	0.674	0.001	0.941	0.46	0.008	0.570	0.723	244.51	**0.001**	92.56	**0.001**
Residuals	273					0.043			0.080			0.736						

Significant *p*-values are highlighted in bold

The total number of alleles per population ranged from 22 in *A. zaliosus* in CL Apoyo to 55 in *A. citrinellus* in L Managua. The number of private alleles per population ranged from 0 in *A. cf. labiatus* in CL Masaya to 19 in *A. citrinellus* in L Managua. At least 9 supertypes were present in each population (Table 3). The total number of alleles per lake ranged from 27 in CL Asososca León to 68 in CL Xiloá, and the number of private alleles ranged from 3 in CL Masaya to 22 in L Managua. At least 10 supertypes were present in the Midas cichlid community of each lake (Table 3). The number of alleles, the number of supertypes, and the number of private alleles were all independent of sample size both at the level of populations and lakes.

**Fig. 4:**
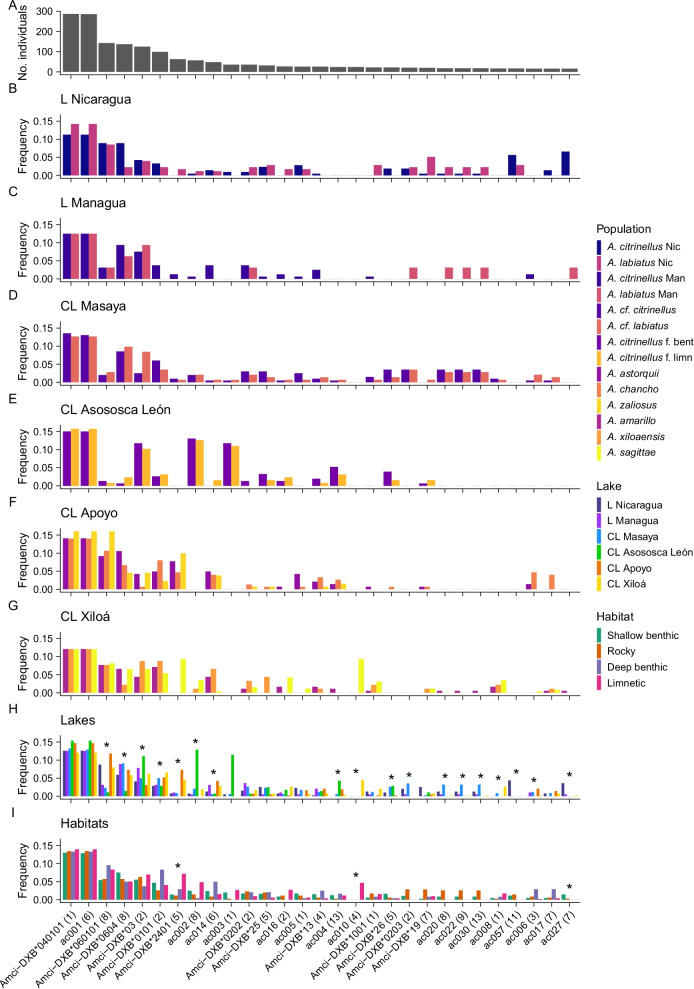
Distribution of MHC IIB alleles present in > 5% of individuals. **A** Number of individuals carrying an allele in the entire data set, **B**–**G** frequency of the alleles within populations split by lake, **H** frequency of alleles within lakes and **I** habitats. Alleles labelled with asterisks (*) contributed to allele pool differences among groups. The supertype that an allele was assigned to is given in parenthesis. Bent = benthic, limn = limnetic

Twenty-nine alleles out of 150 occurred in at least 5% of individuals (Fig. 4A), and were considered as common alleles. Forty-six were singleton alleles. One allele, Amci-DXB*040101, was present in all individuals, and another allele, ac001, was present in all but one. Common alleles varied greatly in frequency among populations (Fig. 4B–G) and cichlid communities of each lake (Fig. 4H). Five alleles were present in all populations and 11 were present in all lake communities. Each supertype was present in at least 26 individuals (ca. 10%). Supertype 1, consisting of 29 alleles, was found in all individuals, and supertype 6, with only 7 alleles, was found in all but one. Supertype 2 (6 alleles) and supertype 8 (4 alleles) were found in 220 (77%) and 255 (89%) individuals, respectively (Additional file 1: Fig. S4A). One supertype was absent in fish from CL Masaya (12), two in fish from CL Apoyo (9, 11), and three were absent in fish from CL Asososca León (10, 11, 12; Additional file 1: Fig. S4C).

**Fig. 5:**
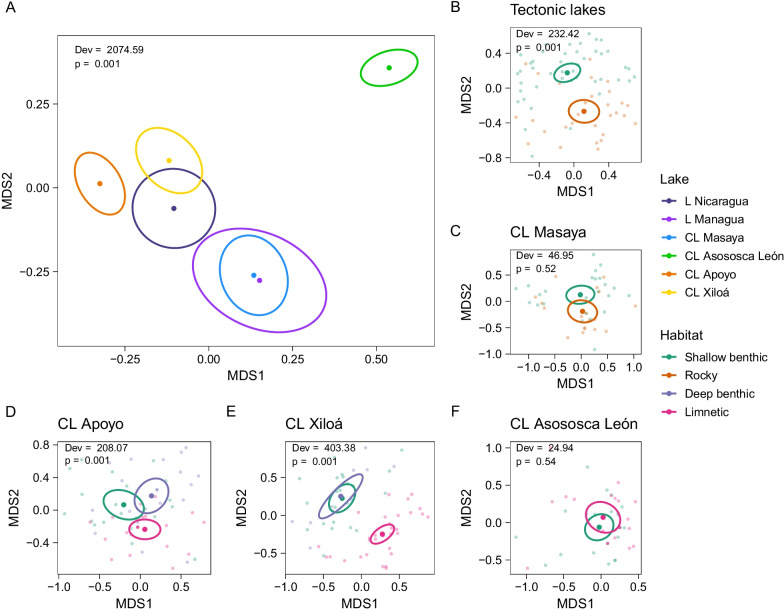
NMDS plots of MHC IIB allele pools. First and second MDS are shown for **A** all individuals grouped by lake, **B** the tectonic lakes Nicaragua and Managua grouped by habitat, and **C**–**F** the crater lakes grouped by habitat. Group centroids (larger points) and 95% confidence ellipses are indicated. Deviances (Dev) and p-values of the multivariate GLMs are provided. Light-coloured points in **B**–**F** correspond to individuals

Allele pools differed among populations (Table 4). This was due to 26 alleles that differed in frequency, 20 of them being common alleles (Fig. 4B–G). The frequency of all supertypes also differed among populations (Table 4, Additional file 1: Fig. S4B). Allele and supertype pools further differed among fish in different lakes, and fish in different habitats across lakes, and the interaction term was also significant (Table 4). Thirty-three alleles (18 being common alleles; Fig. 4H) and 8 supertypes (Additional file 1: Fig. S4C) differed in abundance in fish from different lakes, while 4 alleles (3 of them common; Fig. 4I) and 1 supertype (Additional file 1: Fig. S4D) showed different abundances among fish living in different habitats. Alleles and supertypes that differentiated fish from different habitats also contributed to differentiation of populations among lakes. Pairwise comparisons indicated that, averaged over habitats, fish in each lake had distinct allele and supertype pools that differed from those of fish in all other lakes (Fig. 5A, Additional file 1: Fig. S5). Similarly, allele and supertype pools of all fish exploiting a specific habitat were distinct when averaged over lakes. A codon usage analysis at PSS indicated that MHC alleles between populations in a specific habitat were more similar than expected with shared ancestry (91.6–96.1% observed vs 86.1–90.0% expected identical codons, all p-values < 0.001) and they were highly unlikely to have arisen by convergent evolution (60.3–65.3% expected identical codons, all p-values < 0.001). Within lakes, populations exploiting different habitats differed in their respective allele pools in the large lakes (Fig. 5B) and in crater lakes Apoyo (Fig. 5D) and Xiloá (Fig. 5E), but not in crater lakes Masaya (Fig. 5C) and Asososca León (Fig. 5F).

## Discussion

In this study we investigated the potential contribution of MHC class IIB variation to diversification in the Neotropical crater lake Midas cichlid radiation, one of the most convincing cases of sympatric parallel speciation [51, 52, 61]. We report extensive allelic and also putative functional diversity within and among Midas cichlid populations. Fish from different lakes showed divergent MHC IIB allele pools, as did fish exploiting different habitats within lakes, although no clear parallelism was found. High MHC IIB diversity in the Midas cichlid in particular [47], and in cichlids in general [45], may facilitate the propensity to speciate in this group, as it may facilitate differential local responses and niche specialization upon colonizing new habitats.

The large allelic diversity detected in the Midas cichlid resulted in most alleles being rare, and each occurring only in few individuals. Less than 20% of the alleles were detected in at least 5% of the individuals. Of the 29 most common alleles, eleven were present in fish from all lakes, and only five were recovered from all populations. Since antigen specificity of MHC alleles is predominantly determined by the antigen-binding sites rather than the full coding sequence [62], we clustered alleles into functional supertypes of putatively similar specificity [63]. The 150 alleles recovered in the Midas cichlid converged into 13 supertypes. All supertypes were fairly common across populations, and in each population at least nine were recovered. This suggests that maintaining diverse functionality is relevant for coping with parasite infections, but which variant provides the function may be secondary. This is in line with the hypothesis that balancing selection acts to maintain functional supertypes with rapid turn-over of alleles within supertypes due to arms race dynamics which is supported by a simulation study [64]. Balancing selection on supertypes rather than on alleles can therefore explain why populations and species tend to be markedly differentiated at MHC alleles despite the commonly observed pattern of lineage and supertype sharing. Codon usage indicated that the pattern of lineage sharing was indeed due to shared ancestry and is not an artefact of convergent evolution on sites involved in antigen binding. The importance of functional rather than sequence diversity has also been proposed for other fish species [64, 65], amphibians [66], birds [67], and mammals [68].

We detected lake-specific signatures in the Midas cichlid MHC IIB diversity, suggesting that either phylogenetic ancestry and demographic history have shaped immunogenetic diversity, or within lake characteristics are driving divergent selection among them. Midas cichlid species and populations are more closely related within lakes than among lakes [51, 54, 69], and MHC IIB evolution may at least partially be governed by neutral processes due to colonization patterns. Evidence for neutral evolution at MHC has been described in different systems and at different scales [70, 71]. MHC allele composition of fish in CL Masaya, although differentiated, resembled more that of fish in the great lakes than in any other crater lake. This pattern is also observed for neutral genetic variation and morphological divergence and suggests more recent connections or faunal exchange [61]. On the other extreme CL Asososca León harbours the most genetically differentiated population of all lakes [51, 54], and the most distinct MHC IIB signature was observed for fish of this lake. Also, fish from CL Apoyo and from CL Xiloá each show unique MHC IIB signatures, in line with clear genome-wide divergence [51, 54]. Similarly, populations in the isolated crater lakes Asososca León and Apoyo, that suffered from marked founder effects and/or bottlenecks during their colonization history [51], have eroded MHC diversity. Notably, three widespread supertypes that are present in individuals of all other lakes, are missing in fish from CL Asososca León and two are missing in fish from CL Apoyo. Also, the total number of alleles and functional supertypes per population and the number of alleles and supertypes per individual were lowest within these two lakes. Indeed, population bottlenecks were repeatedly shown to cause severe reductions of MHC diversity that may exceed those at neutrally evolving loci [72, 73]. However, we did not observe a reduction in other sequence diversity parameters, suggesting that to some degree selection has maintained MHC diversity.

Divergent MHC IIB signatures between fish inhabiting different lakes may also be a result of lake features affecting entire species flocks. Abiotic parameters, such as salinity or nutrient levels differ considerably among the Nicaraguan lakes [74, 75]. These can affect parasite communities and may consequently select for different sets of MHC alleles and supertypes among fish inhabiting different lakes. Furthermore, the isolated crater lakes Asososca León and Apoyo harbour an impoverished fish fauna [51, 75] and fewer parasite taxa (Santacruz et al., [44]) compared to the more connected crater lakes Masaya and Xiloá. This difference in fish and parasite diversity is paralleled by differences in MHC IIB diversity among fish inhabiting these lakes. Reduced MHC diversity in populations exposed to less diverse parasite communities is well documented in the well-studied three-spined stickleback [32, 71], and this may also apply to Midas cichlid populations.

Ecomorphologically similar Midas cichlid species assemblages have evolved among lakes providing similar habitat structures [51, 61, 76], despite the unique properties of each lake. Accordingly, we found an MHC IIB signature linked to habitat type, although it was less pronounced than the lake-specific signature. This pattern is the result of divergence from an ancestral allele pool, rather than convergence, consistent with the colonization history and the young age of the Midas cichlid radiations. Notably, limnetic fish had higher genetic diversity per individual than their benthic counterparts when averaged across lakes. Furthermore, allele pools in the deeper crater lakes Apoyo and Xiloá were most divergent between benthic and limnetic species. This is not fully consistent with the phylogenetic relationships in these two lakes which provide evidence that the limnetic species is most distantly related to the benthic species in CL Apoyo but not in CL Xiloá [54, 76]. Allele pools also diverged between *A. citrinellus* and *A. labiatus* that inhabit sandy and rocky substrates in the great lakes, respectively. On the other hand, MHC IIB allele pools were not differentiated between morphotypes in crater lakes Masaya and Asososca León. In these lakes ecomorphotypes are not considered different species, and rather form single polymorphic populations within each lake [61]. Divergence in habitat use goes along with divergence in a number of characteristics that have the potential to alter selection on MHC genes. Parasite communities often differ among habitats, lake and stream, benthic and limnetic, as reported in several freshwater fish species (sticklebacks, whitefish or African cichlids; [18, 40, 77, 78]). Also, different feeding preferences were shown to influence exposure to divergent trophically transmitted parasites in freshwater fish [79–82]. The Midas cichlid species may therefore encounter contrasting parasite communities along the different habitats and prey items they have specialized on. In African cichlids, feeding strategies were shown to affect both MHC allele pool and parasite community compositions [42]. Furthermore, the gut microbiota of the Midas cichlid may have diverged among the benthic-limnetic axis and with trophic ecology within lakes, although with limited parallelism among lakes ([83, 84] but see [85]). Commensal and symbiotic microbial diversity was found to be associated with MHC diversity across the vertebrate clade [27, 28, 86, 87], hence it is conceivable that the microbiota may also exert selective pressure on the Midas cichlid MHC.

Despite the divergence of MHC IIB among habitats, distinct sets of alleles and supertypes were involved in the divergence of populations within each lake. This suggests that differences in selection pressure among habitats may not follow a fully parallel pattern among lakes. An absence of parallelism in MHC allele pools was also reported for whitefish, in which it was associated with a corresponding non-parallelism in microbial pathogens [34]. Information on parasite and microbial infection patterns in the Midas cichlid can elucidate the extent to which such intimate species interactions shape divergence, assortative mating, and speciation in this system.

## Conclusions

Our results provide evidence for substantial immunogenetic divergence among allopatric populations and between habitats among sympatric populations in the Midas cichlid species complex. Despite indication of habitat-specific signatures on MHC IIB, we do not recover clear patterns of parallel divergent selection in ecomorphologically similar species. This suggests that colonization and demographic processes may have shaped MHC evolution along with natural selection.

## Methods

### Sampling

Samples of the Midas cichlid radiation were collected from the two great Nicaraguan lakes, Nicaragua and Managua, and four crater lakes, Apoyo, Asososca León, Masaya, and Xiloá, in December 2009 and 2010. We collected approximately 20 individuals per species or described morphotype inhabiting clearly distinct habitats within each of the six lakes (Fig. 1, Table 1). Fish were caught with gill nets and anaesthetised with Tricaine mesylate (MS-222). Fish were photographed for species and morphotype identification and euthanised on ice. Fin clips were preserved in 100% ethanol for DNA analysis. All methods were carried out in accordance with current Spanish and European Union laws (ECC/566/2015 and 2010/63/UE, respectively) and with the permission of the Ministerio del Ambiente y los Recursos Naturales Nicaragua (MARENA; permit number 001-012012). The study is reported in accordance to ARRIVE guidelines.

### DNA extraction and sequencing

DNA was extracted with the DNeasy Blood and Tissue Kit (Qiagen, Hilden, Germany) following the manufacturer’s recommendations with RNase digestion. DNA concentrations were measured with a NanoDrop 1000 (Thermo Fisher Scientific, Bonn, Germany), standardized to 20 ng/μl, and re-quantified with Qubit (Thermo Fisher Scientific).

For PCR amplification of MHC class IIB exon 2, the forward primer AcMHCIIBF5 (5ʹ CCACKGAGCTGAASGACATSGAG 3ʹ) was used that discriminates against the putatively non-classical alleles [47]. Since the 3ʹ-end of exon 2 was not conserved enough for a single primer to recover all previously characterized *Amphilophus* alleles, two reverse primers were newly designed (AcMHCIIBR10, 5ʹ GCAGTAYNTCYCCYTCTGAG 3ʹ and AcMHCIIBR11 5ʹ GCAGWMTSTCTCCTTYKCAG 3ʹ). These new primers are designed to recover a 142 bp fragment of exon 2 of all but two rare alleles of the known *Amphilophus* alleles.

PCR amplification, library preparation, and amplicon sequencing were done at LGC Genomics (Berlin, Germany). In short, PCR was done in a volume of 20 μl using the MHC-specific primers, tailed with 20 bp derived from Illumina adapters. The mix consisted of 1× MyTaq buffer containing 1.5 U MyTaq polymerase (Bioline, Luckenwalde, Germany), 15 pmol of forward and reverse primers, 2 μl of BioStabII PCR Enhancer (Sigma-Aldrich, Taufkirchen, Germany), and 10–80 ng of DNA. The cycling parameters consisted of 2 min denaturation at 96 °C followed by 20 cycles of 96 °C for 15 s, 50 °C for 30 s, 70 °C for 60 s. Primers were removed with ExoI digestion (New England Biolabs, Ipswich, USA). For library preparation, a second round of PCRs was done with dual index combinations of full-length Illumina adaptors. PCR products were pooled and purified using 1 vol AgencourtXP beads (Agilent) and used in an emulsion PCR with standard Illumina primers using buffers and enzymes from the emPCR Kit (Roche 454) and the oil-surfactant-mixture from the Micellula DNA Emulsion & Purification Kit (EURx). The emulsion was broken and DNA purified following the instructions of the Micellula DNA Emulsion & Purification Kit. A final size selection was done on an LMP Agarose gel. PCR products were sequenced on an Illumina MiSeq V3 resulting in approximately 5 M paired-end reads of 2 × 300 bp.

### Data processing and allele calling

Libraries were demultiplexed with bcl2fastq v1.8.4 (Illumina Inc.), allowing up to two mismatches per barcode. Paired reads with incomplete or conflicting barcodes were discarded, as were reads shorter than 100 bases and those containing more than one N. Remaining reads were trimmed at the 3ʹ end to achieve an average Phred score ≥ 20 over a window of 10 bases. For primer clipping, up to three mismatches were allowed per primer and primer pair. Sequences that did not contain forward and reverse primers were discarded. Primer-clipped forward and reverse read pairs were merged with BBmerge v34.48 [88].

Alleles were called using the command line version of AmpliSAS [89]. Reads were clustered with default parameters for Illumina technology. Per amplicon frequencies (PAF) of clusters, sorted in descending order, were inspected for a distinct drop in frequency which would indicate an optimal PAF [90]. In most samples, this drop occurred between 1 and 2%. PAF (min_amplicon_seq_frequency) was then set to 1.5% which gave a consistent genotype for the sample that was amplified and sequenced in duplicate. The maximum number of expected alleles per individual was set to 50. *Amphilophus* alleles identified by Hofmann et al. [47] were supplied as reference. Alleles newly identified in this study were numbered continuously starting with ac001.

This approach led to somewhat lower numbers of alleles per individual than previously described for *Amphilophus* [47]. Alleles may differ outside the sequenced fragment, and the more stringent filtering approach in this study may collapse alleles differing in only few bases. Previously identified alleles that were grouped together in the current study mostly differed only in synonymous substitutions or < 2 amino acids in the entire exon 2. Since our main focus lies on detecting signatures of divergence, this represents a conservative approach. Also, such high sequence similarity likely represents functional similarity [63] and will only have a minor impact on supertype inference.

### Molecular sequence analyses

Alleles were aligned and variable sites were identified for nucleotide and translated amino acid sequences. Amino acid variability was visualized with a sequence motive logo using the R package ggseqlogo v0.1 [91]. Overall nucleotide p-distance (π), overall amino acid p-distance (aa p-dist), and the overall number of non-synonymous substitutions per non-synonymous site (dN) and synonymous substitutions per synonymous site (dS) were calculated in MEGA X [92]. A gamma distribution with a site-rate parameter of 0.39 was assumed, as indicated by the best-fit substitution model determined with MEGA X. For dN and dS, the Nei–Gojobori method with Jukes–Cantor correction was used. Variance was estimated with 9999 bootstrap replicates.

The phylogenetic relationship among alleles was inferred with a split network constructed in SplitsTree5 v5.0.0 alpha [93] using the Jukes–Cantor method to calculate the distance matrix. The phylogenetic relationship was further determined with Bayesian inference implemented in MrBayes v3.2.7a [94] using codon substitution models. The ω parameter was set to the Ny98 model which allows ω to vary among codons and identifies positively selected sites. Four independent runs with eight chains each with a heating parameter of 0.1 and two swaps per cycle were run for 5 × 10^6^ generations and sampled every 1000 generations. The first 25% were discarded as burn-in. Default values were used for the remaining parameters. Convergence among runs was examined with the R package RWTY v1.0.2 [95]. Majority consensus trees were visualized in R [96].

We estimated historical positive selection over evolutionary timescales on MHC alleles based on ratios of non-synonymous to synonymous substitutions among all alleles. Analyses were conducted on all sites. Gene-wide positive selection on alleles, i.e. along all codons (sites) of all alleles, was estimated with a codon-based Z-test using the Nei–Gojobori distance model with Jukes–Cantor correction and partial deletion in MEGA X. Variance estimation was done with 9999 bootstrap replicates. Gene-wide episodic positive selection was estimated for the full phylogeny with the branch-site unrestricted statistical test for episodic diversification (BUSTED; [97]) available on the datamonkey server [98]. BUSTED tests whether at least one site on at least one branch of the allele phylogeny has evolved under positive selection. Site-specific positive selection was estimated with CodeML implemented in PAML v4.9j [99], the mixed effects model of evolution (MEME; [100]) on the datamonkey server, and the Ny98 model in MrBayes (see above). For CodeML, positive selection was identified by comparing models M1a (nearly neutral, 0 < ω_0_ < 1, ω_1_ = 1) and M2a (positive selection, 0 < ω_0_ < 1, ω_1_ = 1, ω_2_ > 1) and models M7 (10 ω classes following a β distribution) and M8 (β + ω > 1) using codon frequencies estimated from a F3 × 4 nucleotide frequency model. One ω ratio was used for all branches. The codon of amino acid site 41 was excluded due to a deletion in one allele. Likelihood ratio test were used to compare the respective models and PSS were identified with the Bayes empirical Bayes method. The majority consensus tree from MrBayes was used as starting tree. Sites identified by at least two methods were considered to be under positive selection.

Alleles were clustered into functional supertypes following Sepil et al. [101]. For this, 5 z-scores describing the physicochemical properties of amino acids [102] were assigned to each position of unique sequences of concatenated PSS. The resulting matrix was used for discriminant analysis of principle components (DAPC) implemented in the R package adegenet [103, 104]. The optimal number of clusters was identified by K-means clustering using the find.cluster function with the “goodfit” selection criterion. Automatic cross-validation (xvalDapc function) was used for the DAPC. Alleles were assigned to supertypes based on consensus of 5 × 10 clusterings.

### Population divergence analyses

The total number of alleles, private alleles, and supertypes were identified for each population and for each lake. The number of alleles and supertypes per individual was counted. Four additional within-individual sequence diversity indices were calculated in MEGA X [92] using the same parameters as for estimating overall sequence diversity (see above): π, aa p-dist, dN, and dS. Statistical analyses were done in R [96]. Within-individual sequence diversity indices were compared among populations and among lakes, habitats, and their interaction with generalized linear models (GLM). For comparing the number of alleles and the number of supertypes, a quasipoisson distribution with a log link function was used and significance was inferred with Χ^2^ tests. For comparisons involving all other diversity indices, linear models (LM) were used. Post hoc pairwise comparisons using Tukey’s HSD and custom comparisons were done with the emmeans package v1.4.7 [105].

Alleles present in at least 5% of individuals and singleton alleles were identified. For alleles present in > 5% of individuals, frequencies per population and per lake were calculated. To assess whether the allelic composition and the supertype composition of individuals differ among populations and among lakes and habitats, multivariate GLM were performed using the function manyglm of the mvabund package v4.1.3 [106]. Likelihood-ratio-tests were used to evaluate the significance of model terms. Alleles contributing to the difference between groups were identified from the models by reporting univariate statistics with adjusted p-values. Differences in allelic composition were assessed on the full data set. Pairwise multivariate GLM were done to identify which lakes and habitats differ from each other. Benjamini–Hochberg adjustment of p-values was used to account for multiple testing. GLMs were used to assess if populations within tectonic lakes and within each crater lake segregate by habitat based on allelic composition. For lakes with > 2 habitats, this was followed by pairwise multivariate GLM. Segregation of allelic composition was visualized with nonmetric multidimensional scaling (NMDS) based on Jaccard dissimilarities using the vegan package v2.5-6 [107]. Six dimensions were chosen which resulted in a stress close to 0.1. A minimum of 30 and a maximum of 75 random starts were performed, each with a maximum of 1000 iterations. NMDS were calculated for all lakes combined and separately for the tectonic lakes and each crater lake. Four to five dimensions were chosen for single lake analyses.

For populations exploiting the same habitat in different lakes, a codon usage analysis was done with custom scripts provided by Lenz et al. [108] to infer whether co-ancestry or convergent evolution is the most likely scenario for sharing of similar alleles. In brief, for each population pair the number of identical amino acids at PSS was counted between all allele pairs, recording whether they were coded by the same codon. The number of identical codons was compared to a theoretical distribution of expected identical codons under the convergent evolution scenario. This distribution was obtained with 1000 Monte Carlo simulations based on observed codon frequencies of the full sequence in both populations and the observed number of identical amino acids at PSS. Similarly, a theoretical distribution of expected identical codons was obtained for the co-ancestry scenario. Proportion tests were used to estimate significance for each scenario.

## Supplementary Information

**Additional file 1: Figure S1. **Alignment of 142 bp of exon 2 of 150 *Amphilophus *MHC class IIB alleles. Conserved sites are shaded in grey. **Figure S2. **Amino acid variability for 47 positions of 150 aligned Midas cichlid MHC class IIB alleles. **Figure S3. **Phylogenetic relationship of *Amphilophus *MHC IIB alleles. **Figure S4. **Distribution of MHC IIB supertypes. **Figure S5. **NMDS plot for all individuals grouped by lake showing the variation among individuals.

## Data Availability

Raw reads were deposited in the NCBI Sequence Read Archive (BioProject PRJNA736256). Allele sequences are available from GenBank (Accession numbers: MZ368756-MZ368841, MZ368843-MZ368885).
