# Supplementary material for: Divergent and non-parallel evolution of MHC IIB in the Neotropical Midas cichlid species complex
Source: BMC Ecol Evol. 2022 Apr 1;22:41. doi: 10.1186/s12862-022-01997-9 (PMC8974093; doi:10.1186/s12862-022-01997-9)

Additional file 1

Divergent and non-parallel evolution of MHC IIB in the Neotropical Midas cichlid species complex

Seraina E. Bracamonte, Melinda J. Hofmann, Carlos Lozano-Martín, Christophe Eizaguirre, Marta Barluenga

Figure S1. MHC IIB sequence alignment……..…………..……………………………………………2
Figure S2. Amino acid sequence logo………………..……………….…………………………………..3
Figure S3. Phylogeny of MHC IIB alleles………………..…………….……………………………….4
Figure S4. Supertype distribution by populations, lakes and habitats…………………..…..……………5
Figure S5. NMDS plot with individual information ………………..……………………………………..6

Figure S1. Alignment of 142 bp of exon 2 of 150 *Amphilophus* MHC class IIB alleles. Conserved sites are shaded in grey.

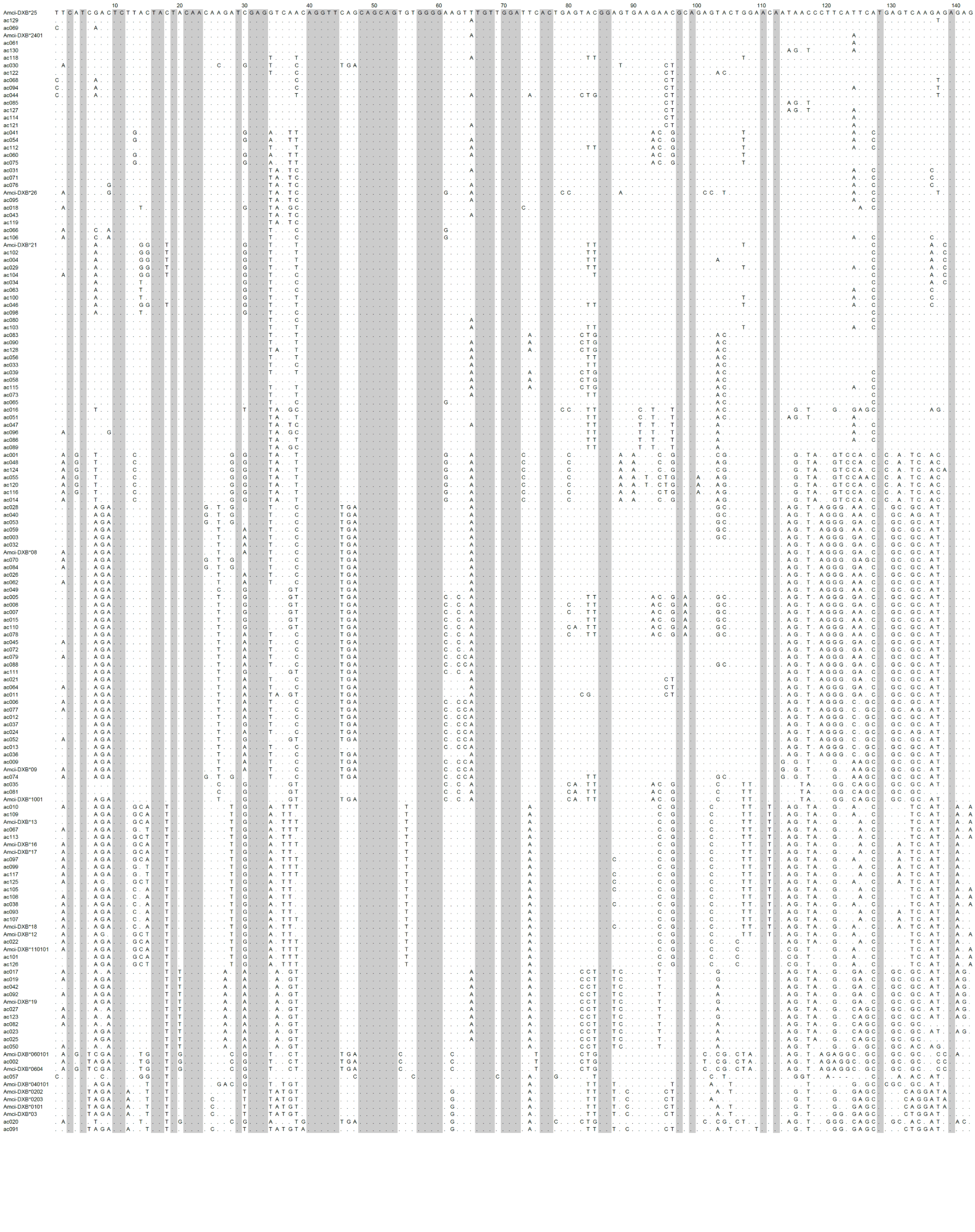

Figure S2. Amino acid variability for 47 positions of 150 aligned Midas cichlid MHC class IIB alleles. Asterisks indicate positively selected sites (** = identified by all methods, * = identified by two methods). Colours indicate chemical properties of amino acids and bits are a measure of information content of each position.

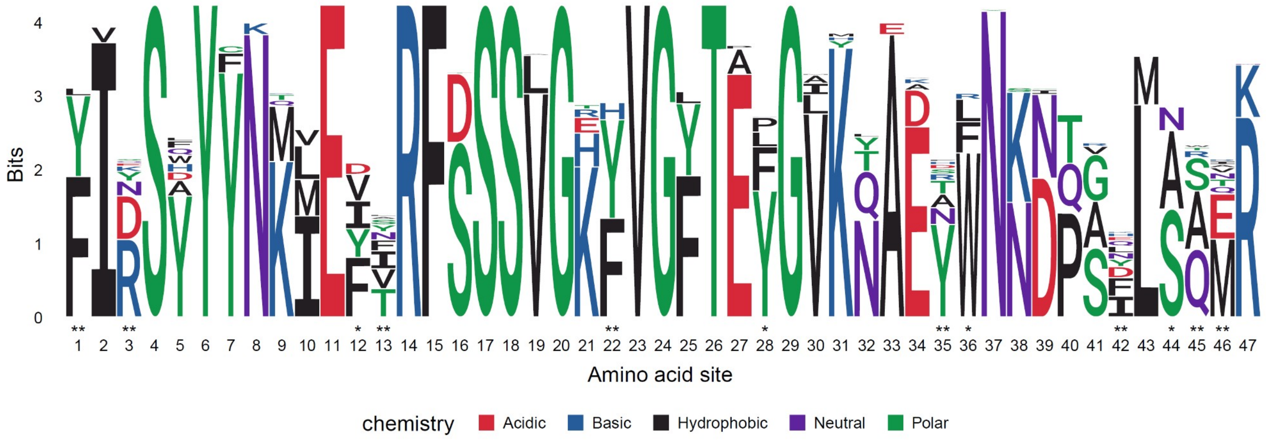

Figure S3. Phylogenetic relationship of *Amphilophus* MHC IIB alleles. Consensus tree from Bayesian inference is shown and splits with posterior probabilities > 50 are indicated.

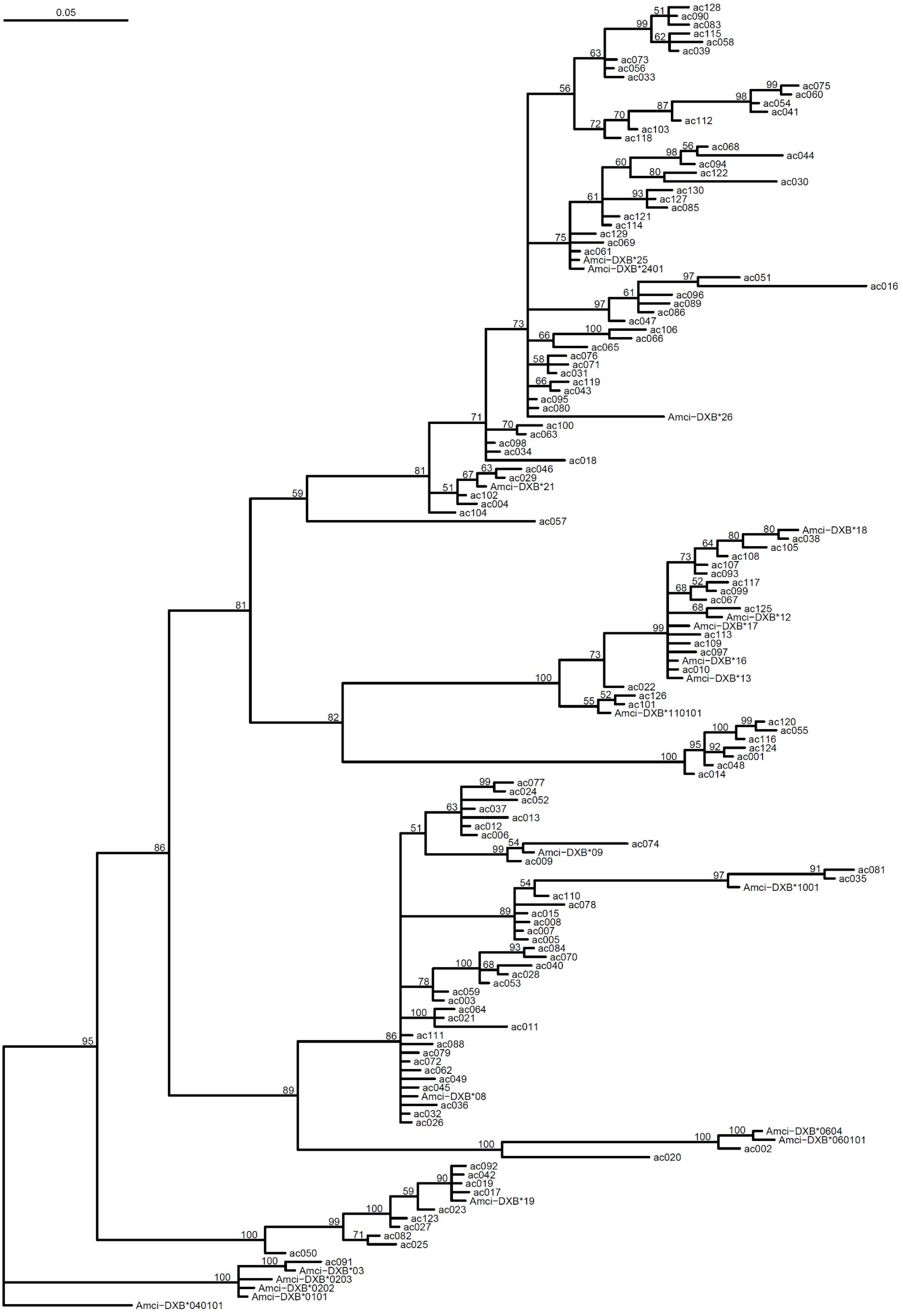

Figure S4. Distribution of MHC IIB supertypes. A) number of individuals carrying a supertype. If multiple alleles of the same supertype occurred in an individual, they were only counted once. B) frequency of supertypes per population, C) per lake and D) per habitat. For supertypes occurring multiple times in an individual, each instance was counted for calculating frequencies in B), C) and D). Numbers in parentheses give the number of alleles per supertype. Asterisks (*) indicate that the supertype contributed to differences among groups. Bent = benthic, limn = limnetic.

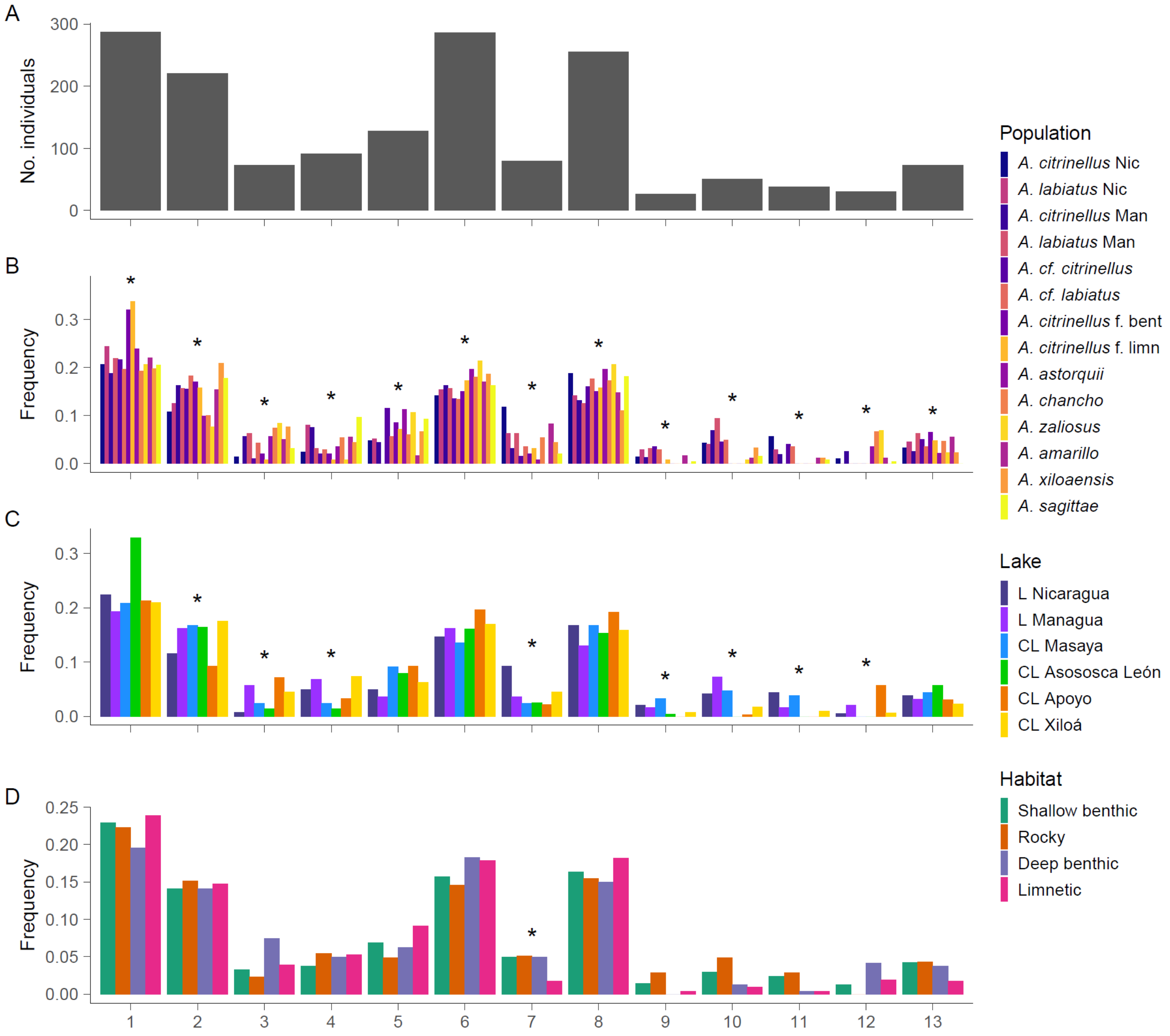

Figure S5. NMDS plot for all individuals grouped by lake showing the variation among individuals. MDS 1 and 2 are shown.

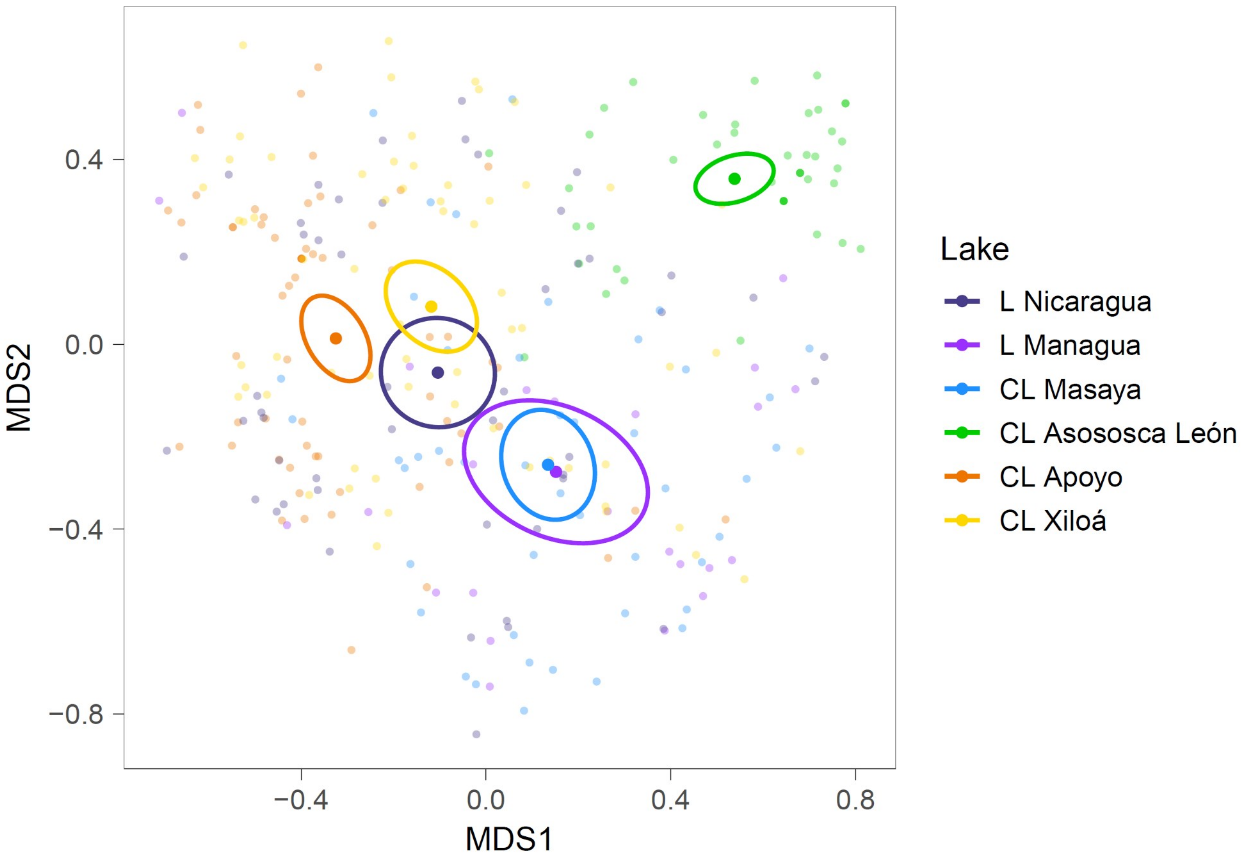

Supplement: Supplementary file 1 — Additional file 1: Figure S1. Alignment of 142 bp of exon 2 of 150 Amphilophus MHC class IIB alleles. Conserved sites are shaded in grey. Figure S2. Amino acid variability for 47 positions of 150 aligned Midas cichlid MHC class IIB alleles. Figure S3. Phylogenetic relationship of Amphilophus MHC IIB alleles. Figure S4. Distribution of MHC IIB supertypes. Figure S5. NMDS plot for all individuals grouped by lake showing the variation among individuals. [file 12862_2022_1997_MOESM1_ESM.pdf]
